# Supplementary material for: Dirac Cones and Room Temperature Polariton Lasing Evidenced in an Organic Honeycomb Lattice
Source: Adv Sci (Weinh). 2024 Apr 12;11(21):2400672. doi: 10.1002/advs.202400672 (PMC11151062; doi:10.1002/advs.202400672)
Supplement: Supplementary file 1 — Supporting Information [file ADVS-11-2400672-s001.pdf]

ADVANCED SCIENCE
Open Access

Supporting Information

for *Adv. Sci.*, DOI 10.1002/advs.202400672

Dirac Cones and Room Temperature Polariton Lasing Evidenced in an Organic Honeycomb Lattice

Simon Betzold, Johannes Düreth, Marco Dusel, Monika Emmerling, Antonina Bieganowska, Jürgen Ohmer, Utz Fischer, Sven Höfling and Sebastian Klembt**

Supporting Information

Dirac Cones and Room Temperature Polariton Lasing Evidenced in an Organic Honeycomb Lattice

Simon Betzold, Johannes Düreth, Marco Dusel, Monika Emmerling, Antonina Bieganowska, Jürgen Ohmer, Utz Fischer, Sven Höfling, and Sebastian Klembt**

S. Betzold, J. Düreth, M. Dusel, M. Emmerling, S. Höfling, S. Klembt

Julius-Maximilians-Universität Würzburg, Physikalisches Institut and Würzburg-Dresden Cluster of Excellence ct.qmat, Lehrstuhl für Technische Physik, Am Hubland, 97074 Würzburg, Germany

A. Bieganowska

Wroclaw University of Science and Technology, Faculty of Fundamental Problems of Technology, Department of Experimental Physics, Wyb. Wyspiańskiego 27, 50-370 Wroclaw, Poland

J. Ohmer, U. Fischer

Julius-Maximilians-Universität Würzburg, Department of Biochemistry, Am Hubland, 97074 Würzburg, Germany

*E-mail: simon.betzold@uni-wuerzburg.de, sebastian.klembt@uni-wuerzburg.de

S1: AFM-measurements of the structured DBR

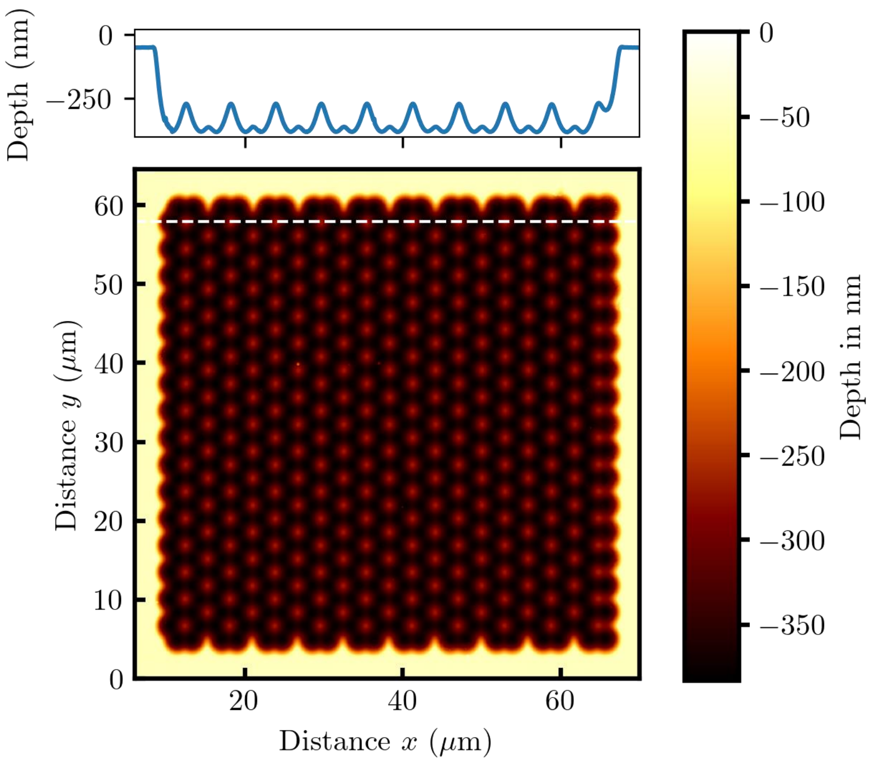

Figure S1. Visualization of the photonic potential landscape illustrated by an AFM image of the honeycomb lattice. The sites have a diameter of 4 µm, a depth of about 380 nm and a center-to-center distance of 2 µm.

S2: Sample assembly

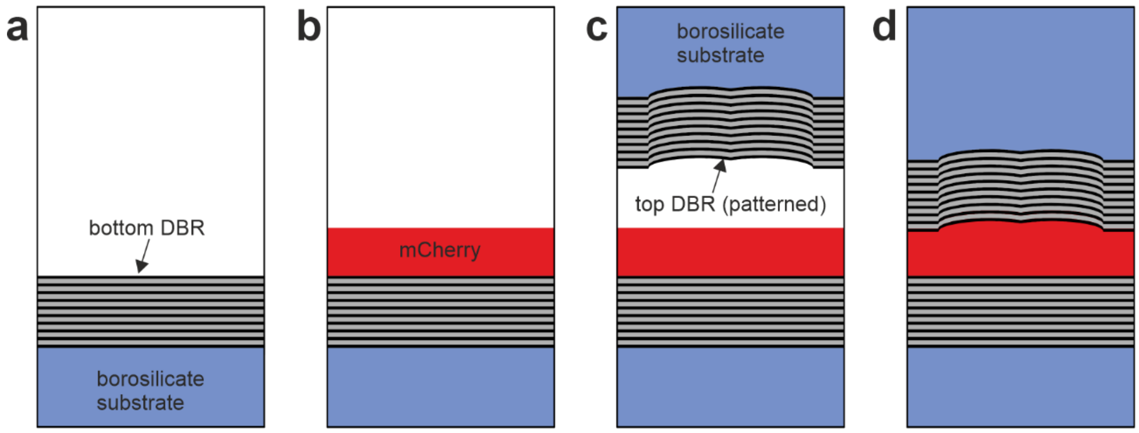

Figure S2. Schematic representation of the sample assembly. Starting with a planar DBR consisting of alternating SiO2/TiO2 layers (a), a solution of mCherry in water is pipetted on top and spread (b). Finally, the second, patterned DBR (c) is applied to the mCherry (d), pressed and left to dry under constant pressure for 48 hours.

S3: Determination of the Rabi splitting, the cavity length, and the Q-factor

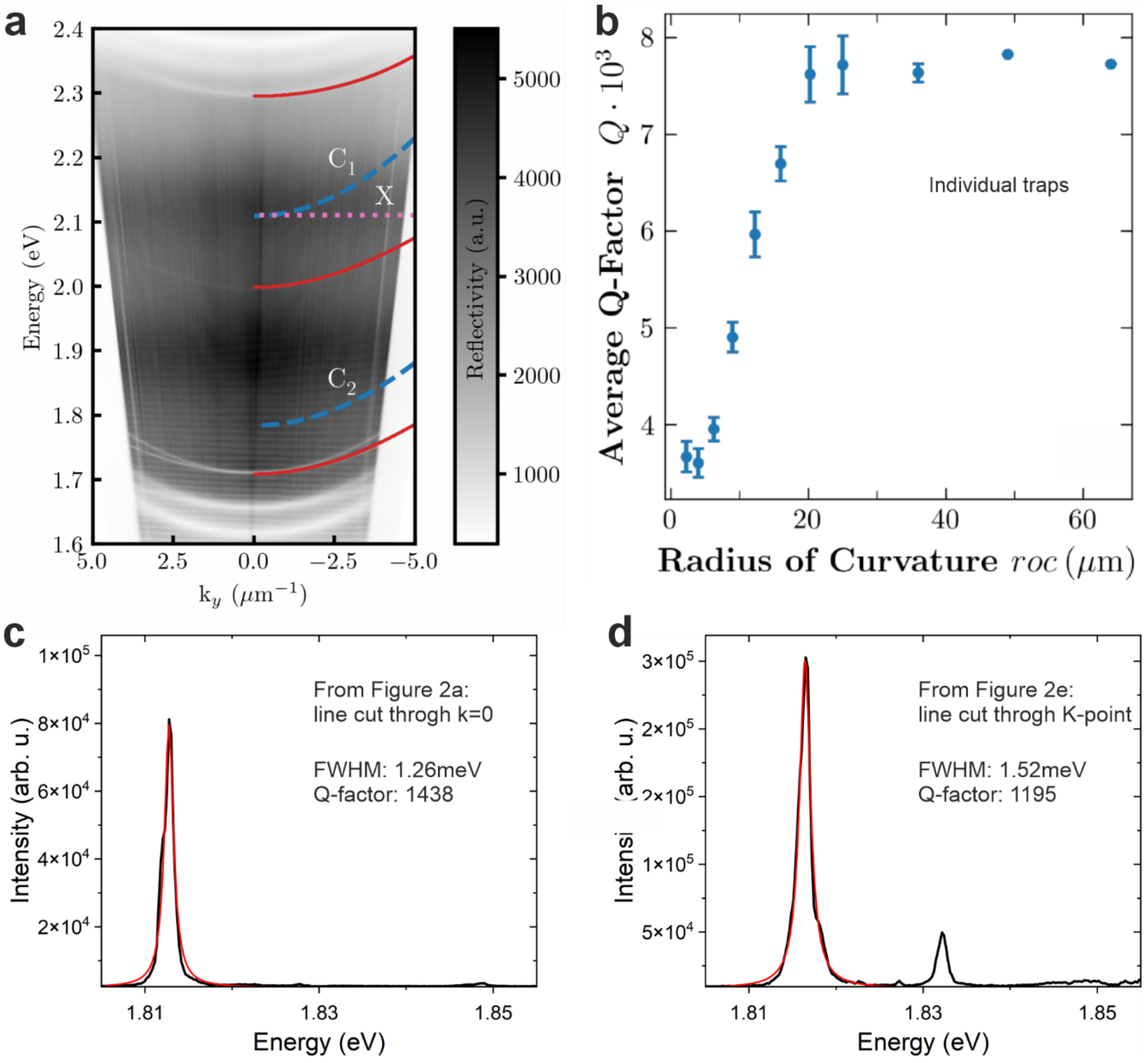

Figure S3: Reflection measurement (a) of the planar cavity near the measured honeycomb lattice of the main text. C1 and C2 denote the photonic modes, while X denotes the excitonic mode. The fitted polaritonic resonances are shown in red. In b) the measured average Q-factor is plotted for potentials with different radii of curvature. Individual hemispheres of the measured honeycomb lattice have a radius of curvature of 10.5 um. c) and d) show spectra taken from line cuts of Figure 2a through k=0 and Figure 2e at the K-point, respectively. The Q-factor of the binding s-band is about 1400, while the Q-factor at the K-point is about 1200.

To determine the cavity length and Rabi splitting of the sample, three independent white light reflection measurements at different detunings were evaluated. The spectral position of the photonic modes C1 and C2 was determined from transfer matrix simulations of the empty cavity

and iteratively updated to find the Rabi splitting at the three positions. Our results yield an average Rabi splitting of (313.8±4.7) meV and optical thicknesses for the mCherry layer of about 1100 nm, 1150 nm, and 1080 nm. As expected, the Rabi splitting is almost constant over this detuning range, so we show the measurement in Figure S3a at an optical thickness of mCherry of about 1100 nm and a Rabi splitting of 318 meV.

We include Figure S3b to illustrate the dependence of the Q-factor on the radius of curvature of the structures. The data shown in this figure are measured on a different sample but using similar mirrors and structures. The Q-factor reaches a maximum of about 8000 for radii of curvature larger than 30 µm but decreases rapidly below this value as scattering losses increase and the stability condition for stable resonators is approached.

In addition, Figure S3c and d show that the Q-factor decreases further in a lattice of coupled resonators due to increased scattering losses.

S4: Mode tomography in the linear regime of the mode analyzed in Figure 3

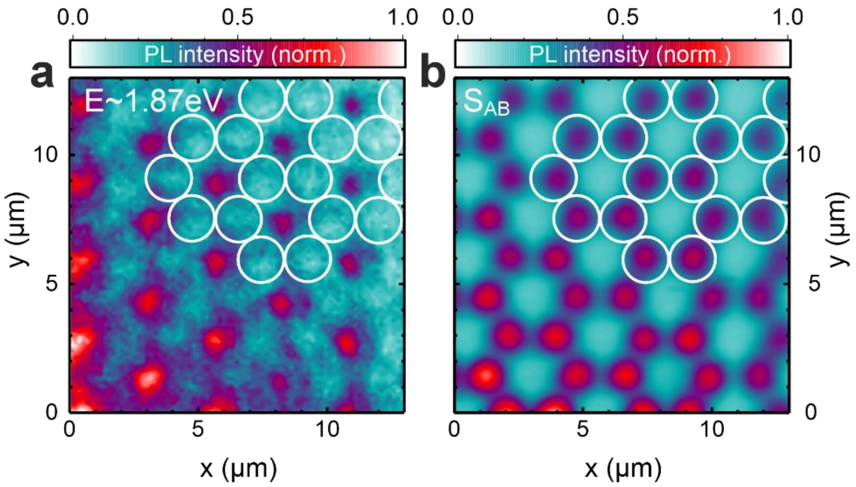

Figure S4: (a) Real-space image, for which the intensity was spectrally summed between 1.869 eV and 1.872 eV. The origin of these specific Bloch mode is most likely due to the interference of the $3d_{xy}$ modes of the individual hemisphere potentials. For this reason, the center of each hexagon shows the highest emission intensity, while the part of the modes located in the center of each hemisphere interferes destructively. A similar signature was observed by Kim et al. in a square lattice in an inorganic microcavity.[56] (b) For comparison: Real-space image of the anti-binding S subband, for which the intensity was spectrally summed between 1.818 eV and 1.830 eV (same as Figure 2h) of the main text).

S5: Additional information about the experimental setup

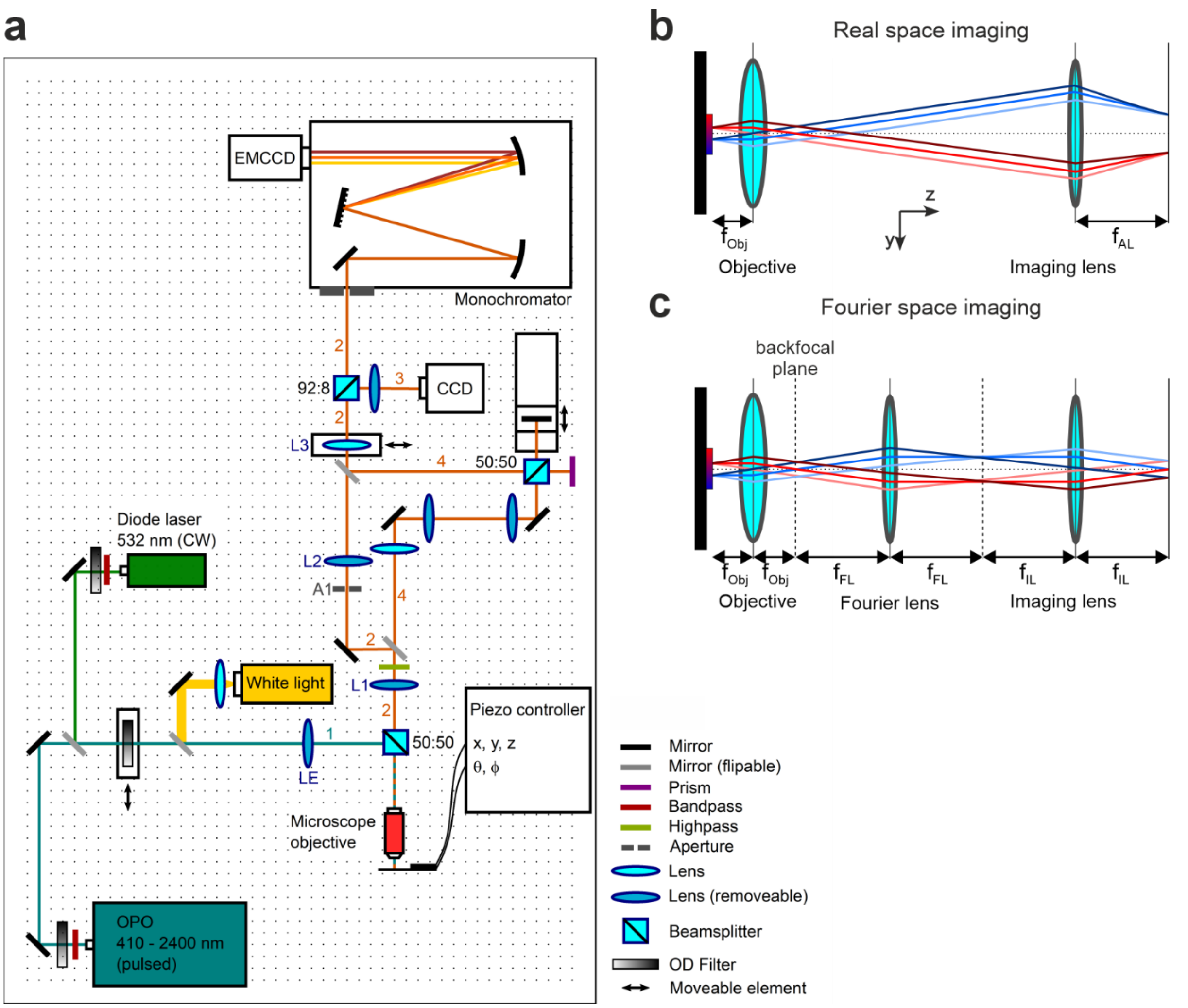

Figure S5. Schematic drawing of the measurement setup with the most important components for photoluminescence and reflection measurements (a) and schematic lens configuration to measure the real space (b) and the Fourier space (c).

Various laser systems are available for measurements, directed through (flip) mirrors onto the excitation path (Figure S5, Path 1). For basic sample characterization, a frequency-doubled ND:YAG laser diode with an emission wavelength of 532 nm (Atelier Rieter, DPSS module, 200 mW output power, CW) is used. Additionally, an optical parametric oscillator (OPO) from

OPOTEC Inc. is available for measurements in the nonlinear regime, tunable from 410 to 2400 nm, with pulse rates up to 20 Hz, a pulse length of 7 ns, and wavelength-dependent output power up to 9 mJ. The excitation power to the sample can be varied using a movable continuous OD filter. A white-light source can also be coupled into the excitation path using a flip mirror. The emission from the excitation sources is directed from the excitation path via a 50:50 beam splitter to a microscope objective with 50x magnification and a numerical aperture of NA=0.42, focusing it onto the sample. A lens (Lens LE) can be introduced into the beam path, allowing measurements with an expanded laser spot. The emitted or reflected light is collected by the same microscope objective, passes through the beam splitter again, and is directed to the detection path (Path 2). A lens system leads to a Czerny-Turner spectrometer (Andor Solis Shamrock 500i), which spectrally filters the signal and directs it to a Peltier-cooled CCD camera. The CCD camera (Andor Newton 971) has an EM sensor and a resolution of 1600 x 400 pixels with a pixel size of 16 µm x 16 µm. This configuration allows a maximum nominal spectral resolution of about 200 µeV in the relevant energy range around 2 eV. Additionally, a second CCD camera without spectral resolution capability is accessible through Path 3.

The lens system in detection Path 2 enables measurements in both real-space and momentum-space (Fourier space). The two measurement methods are schematically depicted in Figure S5b and c. The microscope objective is simplified here as a single lens. For real-space, only Lens L3 from Figure S5a is needed, functioning as an imaging lens and focusing the real-space image onto the entrance slit of the monochromator. The magnification of the real-space image is given by the ratio of the focal length of the imaging lens f_{IL} to the focal length of the objective f_{Obj}. The chip of the CCD camera displays the image of the sample with an open entrance slit and at a grating position in 0th reflection order. Closing the entrance slit and rotating the grating allows energy resolution, reducing the spatial information to the x-direction while $y \approx 0$.

For measurements in momentum-space, the Fourier lens L1 is introduced. Taking advantage of the fact that all rays emitted from the sample at a certain angle converge to a point in the back-focal plane of the objective, Lens L1 and Lens L3 project the Fourier plane onto the entrance slit of the monochromator. Closing the entrance slit to about 30 µm reduces the analyzed light to wave vectors around $k_y \approx 0$. An intermediate real space image is formed between the Fourier lens and the imaging lens, and the aperture A1 in this plane allows selection of the spatial region for the k-resolved measurement.

Lens L3 is mounted on a motorized linear stage, enabling consecutive sections of the real or momentum space at a fixed y or k_y value to be captured and then assembled. This allows, for example, the generation of an energy-resolved image of the real space without limiting it to a

cut in one dimension. This measurement method is referred to as mode tomography in the main text. To provide a conceptual distinction from measurements in momentum space, the term hyperspectral imaging is used here.
A flip mirror in detection path 2 can guide the emitted radiation back into the detection path via a Michelson interferometer (Path 4). This allows measurements of the spatial (and temporal) first-order correlation function.

References

[56] N. Y. Kim, K. Kusudo, C. Wu, N. Masumoto, A. Löffler, S. Höfling, N. Kumada, L. Worschech, A. Forchel, and Y. Yamamoto, Dynamical d-wave condensation of exciton–polaritons in a two-dimensional square-lattice potential, *Nat. Phys.* **2011**, *7*, 681.
